# Early‐Onset Diabetes in Ghana's Upper East Region—Insights From Hospital Data

**DOI:** 10.1002/edm2.70079

**Published:** 2025-07-26

**Authors:** Solomon Beletaa, Ceasar Kaba, Joy Afua Mensah, Gideon Kofi Helegbe, James Abugri, Samuel Mawuli Adadey

**Affiliations:** ^1^ Department of Biochemistry and Molecular Medicine School of Medicine, University for Development Studies Tamale Ghana; ^2^ College of Health Sciences University of Ghana Accra Ghana; ^3^ Department of Biochemistry and Forensic Sciences School of Chemical and Biochemical Sciences, C. K. Tedam University of Technology and Applied Sciences Navrongo Ghana

**Keywords:** diabetes complications, early‐onset diabetes, juvenile diabetes, maturity‐onset diabetes of the young (MODY), monogenic diabetes, type 1 diabetes

## Abstract

**Background:**

Diabetes mellitus is the most prevalent endocrine disorder in individuals under 30 years, encompassing type 1 diabetes mellitus (T1DM), early‐onset type 2 diabetes mellitus (T2DM), monogenic diabetes, and maturity‐onset diabetes of the young (MODY). This study investigated the prevalence, types, and complications associated with early‐onset diabetes (EOD) in the Upper East Region of Ghana.

**Methods:**

The study used anonymised patient data from the Ghana Health Service's electronic data system, focusing on inpatient records of individuals aged 0 to 30 diagnosed with diabetes. After removing personal identifiers, incomplete records, gestational diabetes cases, and duplicates, the dataset included variables such as age, sex, education level, admission year, outcomes, diagnoses, and complications, but lacked laboratory and treatment information.

**Results:**

The prevalence of EOD among patients under 30 years of age was calculated to be 0.16% (52 out of 33,282). T1DM was diagnosed in 15 out of 52 patients (28.8%), while only one case of T2DM was identified. Secondary diabetes due to unknown etiologies was the most common diagnosis (22 out of 52 cases, 42.3%), indicating the potential presence of undiagnosed monogenic diabetes or MODY. Reported complications included diabetic foot (5 cases), diabetic nephropathy (2 cases), infections (4 cases), retinopathy (4 cases), and ketoacidosis (13 cases). The data showed 3 deaths, 1 referral, and 1 absconded case were recorded.

**Conclusion:**

These findings highlight the need for accurate diagnosis, targeted management strategies, and further research into secondary diabetes and its potential underlying causes in Ghana. Improved diagnostic capabilities, awareness, and healthcare resources are essential to address EOD and its complications at the study site.

## Graphical abstract

This graphical abstract illustrates the prevalence, types, and complications of early‐onset diabetes (EOD) among patients under 30 years in Ghana's Upper East Region, based on hospital data. It highlights the predominance of secondary diabetes of unknown cause, associated complications, and the urgent need for improved diagnostic capabilities and targeted management strategies.

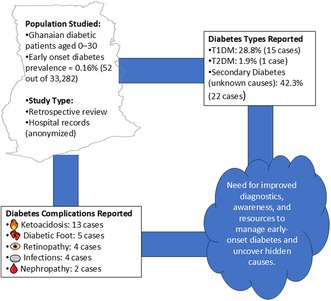

## 1 Introduction

Diabetes mellitus is a chronic metabolic disorder characterised by persistent hyperglycaemia resulting from insufficient beta‐cell function or compromised insulin secretion [1]. In 2021, over 537 million adults globally were estimated to be living with the condition, and the numbers are expected to increase to 643 million by 2030 and 783 million by 2045 [2]. Low‐and middle‐income countries (LMIC) have the highest burden of diabetes (80%) [3]; however, it is estimated that about half (50%) of adults with diabetes living in LMICs are undiagnosed [3]. In Africa, the prevalence of diabetes is increasing, which may be due to rapid urbanisation and unhealthy dietary habits, lifestyle changes, and socioeconomic challenges. Recent data suggested that over 19 million Africans currently live with diabetes, and the number is projected to rise to 47 million by 2045 [4]. Similar to the continental trends, the cases of diabetes in sub‐Saharan Africa are also on the rise, and the prevalence was estimated at 5.7% [5]. However, the prevalence in Ghana is lower among adults, approximately 3.6% [2], although the true burden might be higher due to underreporting and limited diagnostic capacity.

In young people below 30 years, diabetes mellitus is the most common endocrine disorder, with an estimated 35 per 10,000 youth affected in the United States [6]. Diabetes that occurred before 30 years can be considered as early‐onset diabetes (EOD) and comprises type 1 diabetes mellitus (T1DM), early‐onset type 2 diabetes mellitus (T2DM), monogenic diabetes, and maturity‐onset diabetes of the young (MODY). Among the types of diabetes, T1DM accounts for the majority of cases of diabetes mellitus in children, with its incidence peaking between the ages of 10 and 14 years [7]. It is estimated that approximately 15 per 100,000 live births globally are diagnosed with T1DM. T1DM incidence rises by 2%–5% annually, with approximately 200 children being diagnosed with the condition each day [7]. In 2021, an estimated 8.4 million people worldwide were living with T1DM, and 1.5 million (18%) were under the age of 20. Low‐middle‐income countries accounted for one‐fifth of T1DM cases [8].

T1DM is mainly caused by autoimmune destruction of the insulin‐producing cells of the pancreas [9]. The destruction of pancreatic beta cells happens gradually and silently over a period of many years. Clinical symptoms begin to appear when about 80% of pancreatic cells are destroyed [10]. T1DM is diagnosed based on clinical presentation, blood glucose levels, and the presence of autoimmune antibodies, and mostly manifests as polyuria, polydipsia, significant weight loss, or diabetic ketoacidosis (DKA) [1]. These patients have fasting blood glucose levels of greater than 7.0 mmol/L or random blood glucose levels more than 11.0 mmol/L. T1DM diagnosis is confirmed by the presence of antibodies such as glutamic acid decarboxylase antibodies (GADA) and islet cell antibodies (ICA) [1]. Due to the lack of testing facilities and financial constraints, serological confirmatory testing is a significant challenge in most African countries. On the other hand, T2DM is characterised by insulin resistance, which may be combined with a progressive impairment of insulin secretion. It predominantly occurs in adults, but in recent times it is increasingly diagnosed in young people due to the growing prevalence of obesity and sedentary lifestyles [9]. Early‐onset T2DM is a growing global health concern with an onset at a younger age, typically before 30 years. Early‐onset T2DM is associated with a more aggressive disease progression and a higher risk of complications compared to late‐onset diabetes [11]. Monogenic diabetes is caused by single‐gene mutations and accounts for 1%–5% of diabetes cases in children [12], and includes subtypes such as maturity‐onset diabetes of the young (MODY) and neonatal diabetes mellitus (NDM). MODY is the most common form of monogenic diabetes and occurs mostly before age 25, while neonatal diabetes presents within the first six months of life [13]. However, in rare cases, neonatal diabetes manifests as late as 12 or even 24 months of age [14].

Early‐onset diabetes requires treatment with specific drugs and follow‐up with daily multiple insulin injections, a diet with carbohydrate calculation, maintenance of physical activity, and continuous glucose monitoring to achieve control of glycemic blood levels [15]. Patients living with EOD mellitus have a higher risk of developing microvascular and macrovascular diseases, heart failure, and shorter life expectancy compared to late‐onset diabetes [11].

Children with EOD are reported to have poor quality of life (QoL) with frequent hospitalisation, which ultimately affects their academic performance [16]. It is worth noting that greater QoL is associated with better glycaemic control in children with EOD [15]. Serious physical and mental health issues easily occur in children with diabetes due to the early onset age, long durations of hyperglycaemia, multiple complications, and other factors, casting a heavy economic burden on affected families [11]. Also, managing diabetes presents a significant challenge to affected children and their families. Children with diabetes often experience physical, emotional, and social difficulties due to the disease's demanding nature. Strict dietary restrictions, frequent glucose monitoring, and insulin injections can cause distress, particularly in younger children [17]. In Ghana, insulin is only intermittently available at the government healthcare facilities. Blood glucose meters with their strips and haemoglobin A1c (HbA1c) testing are also not easily accessible and are expensive [18]. The financial burden, stigma, and psychological challenges often lead to poor adherence to treatment, which worsens glycaemic control.

Finally, there is a paucity of literature reporting the incidence, distribution, and prevalence of EOD in Ghana. The few reports are from the two major cities, Kumasi and Accra, with no reports from the Upper East Region and other rural settlements. Therefore, we present in this study the socio‐demographic distribution, complications, outcome, and challenges of early‐onset diabetes among patients in the Upper East Region of Ghana using hospital data.

## 2 Methods

### 2.1 Ethical Consideration

Ethical approval was sought and obtained from the Navrongo Health Research Centre, Institutional Review Board (NHRCIRB) (ethics approval ID: NHRCIRB624). Administrative clearance was obtained from the Bolgatanga Regional Hospital, Ghana, to access patient records.

### 2.2 Study Site

Previous studies on EOD in Ghana have primarily focused on the major cities (Accra and Kumasi), with, to the best of our knowledge, no reports from the northern regions. Therefore, the Upper East Region was selected as the study site. The research was conducted at the Bolgatanga Regional Hospital, a tertiary facility that serves as a referral centre for district hospitals and smaller healthcare facilities in the region. Suspected cases of EOD are commonly referred to this hospital due to diagnostic challenges and associated complications. Additionally, many smaller hospitals in the region lack the resources needed to accurately diagnose such cases, further reinforcing the regional hospital's central role in managing them.

### 2.3 Data Collection

Patient data from the Bolgatanga Regional Hospital, recorded in the District Health Information Management System (DHIMS2) of the Ghana Health Service, were collected and utilised for this study. Personal identifiers such as name, national identification number, date of birth, and address were deleted from the data during the data downloading process. Furthermore, the data was thoroughly checked after downloading to ensure that no individual could be reidentified. Inpatient records for individuals diagnosed with diabetes and within the age groups of 0 to 30 were retained for analysis. The data was subsequently cleaned by removing individuals with 50% missing data, people diagnosed with gestational diabetes, and duplicated records. The data elements present were age, sex, level of education, year of hospitalisation, outcome of admission, diagnoses, and complications. The data did not include the laboratory tests conducted and the treatments given.

### 2.4 Statistical Analysis

Since the Chi‐square test is not suitable for data with low counts, and some of the categories in our data have low counts, Fisher's Exact test was used to test the association between diabetic complications and other variables such as age, sex, education, and type of diabetes. P‐values lower than 0.05 were considered significant.

## 3 Results

A total of 33,282 patients less than 30 years old were hospitalised at the study site from January 2021 to April 2024, of which records of 92 patients with a possible diabetes diagnosis were retrieved from the hospital records. After the removal of duplicates, gestational diabetic cases, and other cases that do not qualify as early child onset diabetes, 52 records were retained and analysed. Therefore, among patients younger than 30 years, the prevalence of EOD was calculated at 0.16%, based on 52 cases out of a total of 33,282.

### 3.1 Sociodemographic Characterisation of Participants

**FIGURE 1:**
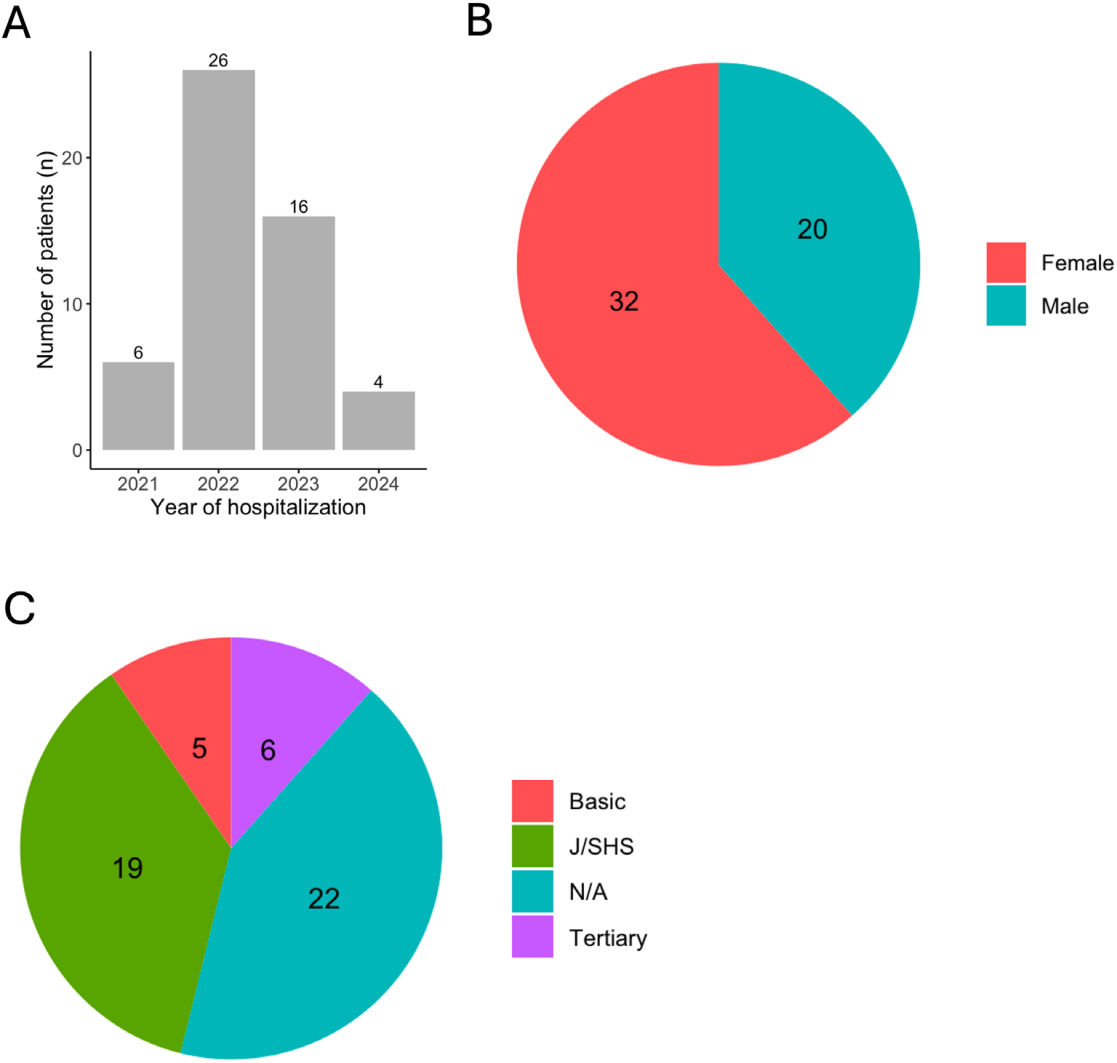
Demographics of study participants. (A) Distribution of year of hospitalisation of early‐onset diabetic cases. (B) Gender of study participants. (C) Educational level of participants. (N/A) The level of education was missing for some participants, and J/SHS denotes junior or senior high school.

**FIGURE 2:**
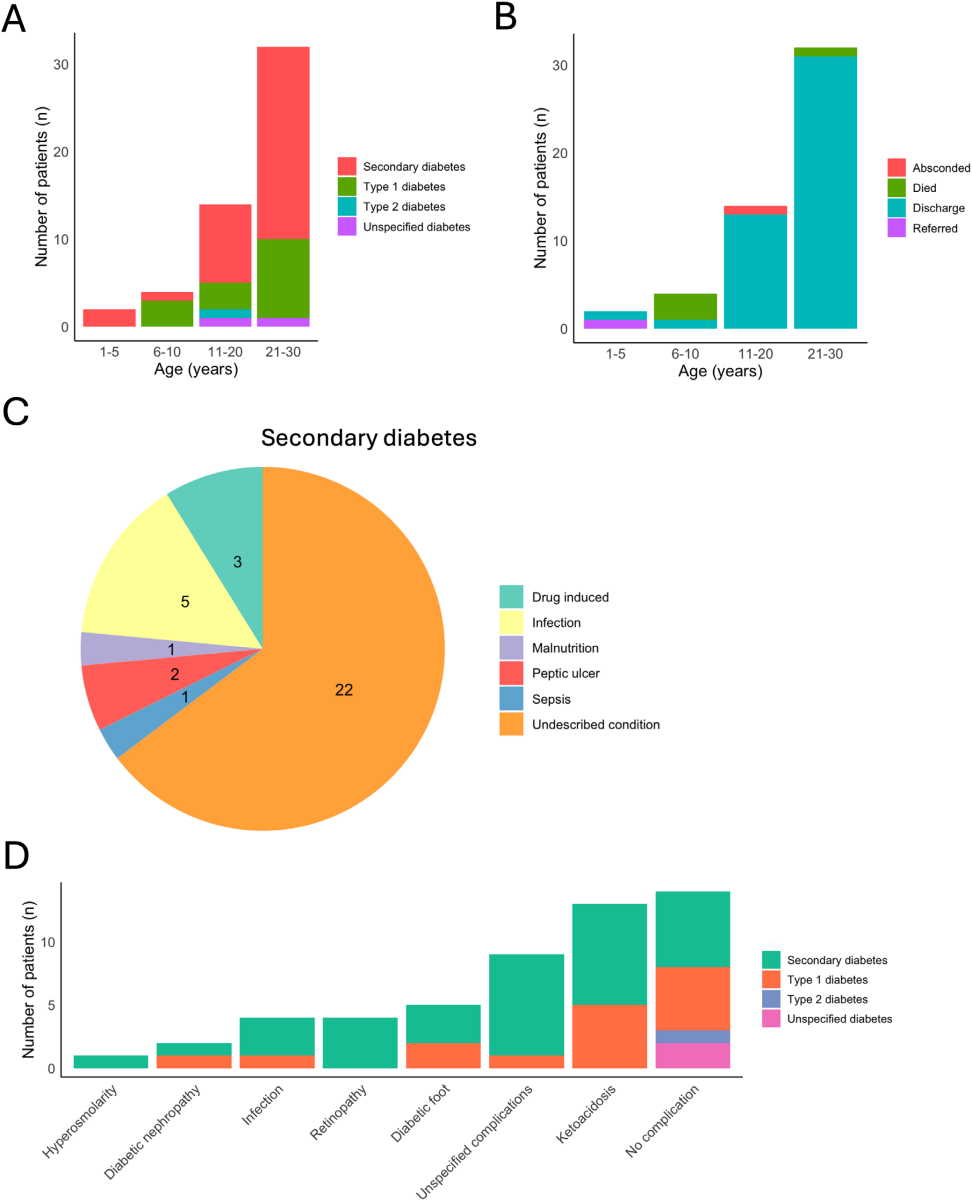
Clinical diagnosis and outcomes of patients. (A) type of diabetic cases diagnosed and categorised into age groups. (B) Clinical outcome of patient hospitalisation. (C) Categories of secondary diabetes diagnosed. (D) Diabetic complications in early‐onset diabetic patients.

The year 2022 recorded the highest (26 out of 52 patients) number of early‐onset diabetic cases (Figure 1A) with most of them being females (32 out of 52 patients). Most patients included in the study were between 21 to 30 years old (Figure 2A). The data reveal that most of the patients are students; 19 were in junior or senior high school, while 6 and 5 of them were in basic and tertiary schools, respectively (Figure 1C).

### 3.2 Types and Trends of Early‐Onset Diabetes in the Upper East Region of Ghana

The data retrieved from the hospital did not include the age at diagnosis; however, all the patients analysed were less than 30 years old, which qualifies them to be classified as early‐onset diabetic cases. Twenty patients (28.4%) were less than 20 years old and may qualify to be classified as juvenile diabetic cases, especially T1D cases. Only one patient reported with type 2 diabetes, and 15 out of 52 patients (28.8%) were diagnosed with T1DM (Figure 2A). Secondary diabetes (22 out of 52 cases, 42.3%) was the most common diagnosis (Figure 2A). In most cases of secondary diabetes, 22 out of 44 patients, the underlying cause of the disease was unknown (Figure 2C).

There were no consistent annual trends in the diagnosis of EOD at the study site. No cases of early‐onset type 1 diabetes (T1DM) were recorded in 2021, while 5, 8, and 2 cases were diagnosed in 2022, 2023, and 2024, respectively. Only one case of early‐onset type 2 diabetes (T2DM) was reported in 2023. For secondary diabetes, 6 cases were recorded in 2021, 19 in 2022, 7 in 2023, and 2 in 2024 (Table 1).

**TABLE 1:** Yearly distribution of the types of early‐onset diabetes.

Year of hospitalisation	Secondary diabetes	Type 1 diabetes	Type 2 diabetes	Unspecified diabetes
2021	6	—	—	—
2022	19	5	—	2
2023	7	8	1	—
2024	2	2	—	—

Among the participants, 14 (26.9) did not present with any diabetic complications. However, several patients did report complications, including diabetic foot (5 patients), diabetic nephropathy (2 patients), infections (4 patients), retinopathy (4 patients), and ketoacidosis (13 patients) (Figure 2D). It is worth noting that almost all the patients were treated and discharged, with 3 deaths, 1 referred, and 1 absconded (Figure 2B).

### 3.3 Factors Associated With Complications Among Early‐Onset Diabetes Patients

In our study, the type of diabetes was significantly associated with the development of complications (*p* < 0.05), indicating that the specific diabetes types significantly influenced the risk of complication development. Secondary diabetes (53.8%) and T1DM (19.2%) were identified as the primary contributors to the development of complications (Table 2).

**TABLE 2:** Association between predictive variables and early onset diabetes complications.

Variable	Category	Complication	No complication	Fisher's Exact Test
Age (years)	1–5	1	1	0.2121
	6–10	4	0	
	11–20	8	6	
	21–30	25	7	
Sex	Female	24	8	0.7542
	Male	14	6	
Education	Basic	3	2	0.757
	J/SHS	13	6	
	Tertiary	5	1	
	N/A	17	5	
Type of diabetes	Secondary diabetes	28	6	0.01576
	T1DM	10	5	
	T2DM	0	1	
	Unspecified diabetes	0	2	

*Note:* Fisher's Exact Test less than 0.05 significance level was considered statistically significant.

## 4 Discussion

Understanding the distribution, clinical presentation, and outcome of early‐onset diabetes is critical in improving awareness and care, especially in low‐ and middle‐income countries (LMIC). Ghana faces significant challenges in the diagnosis and management of early‐onset diabetes. Childhood diabetes cases are often misdiagnosed due to a lack of immunological and genetic testing in most healthcare facilities. This poses a significant threat to the management of diabetes. The various types of early‐onset diabetes are sometimes misdiagnosed as T1DM, leading to inappropriate treatment regimens such as insulin therapy instead of oral sulfonylureas [14]. Diagnostic errors are compounded by the lack of awareness of the rare forms of EOD among healthcare providers and the general population [18]. Misdiagnosis not only delays proper treatment but also increases the risk of long‐term complications, such as growth failure and diabetic ketoacidosis. This study therefore sought to investigate the prevalence and types of early‐onset diabetes diagnosed in the Upper East Region of Ghana.

Analysis of our results revealed that early‐onset diabetes was more common in females (61.5%) compared to males (38.5%). A previous study by Ameyaw et al. (2017) which retrospectively reviewed the clinical features of all children and adolescents with new‐onset diabetes at Komfo Anokye Teaching Hospital in Kumasi, revealed that EOD was more common in females (71%) compared to males (29%), coinciding with our finding [18]. Similar to the studies from Ghana, a Nigerian study reported 65.0% girls with early onset diabetes against 35.0% boys [19] while a 1:1 ratio was reported by another Nigerian study on T1DM [10]. However, more males were found to be living with childhood T1DM in Sweden (53.9%) [20] and Germany (55.8%) [21] which is contrary to our report. Although our analysis shows that gender may not significantly contribute to the development of early onset diabetes, a review published striking differences in the outcome and severity of childhood T1DM based on sex [22]. Girls, especially during adolescence, were found to have poor glycemic control, higher BMI, and severe forms of the disease compared to boys of the same age [22]. These disparities may be explained by lifestyle and hormonal differences between males and females.

Our results indicated that secondary diabetes was the most prevalent form of diabetes (65.4%) among patients with EOD at the study site. Most cases of secondary diabetes had an unknown aetiology (42.3%). This may indicate other undiagnosed cases of monogenic diabetes or MODY. Underdiagnosis or misdiagnosis of monogenic diabetes cases reflects the unavailability of molecular diagnostic facilities in most parts of the country. Misdiagnosis of diabetes has dire consequences for the management of diabetes, as it may lead to suboptimal treatment plans ultimately affecting patient outcomes [14]. Our data indicated that T1DM was more common, 15 (28.8%) among EOD patients compared to T2DM1 (1.9%). Our result agrees with similar studies from Ghana (84.9% T1DM and 15.1% T2DM) [18] and Nigeria (61% T1DM and 28% T2DM) [19]. These findings could be explained by the age of onset of these forms of diabetes; T1DM is noted for early onset, before 30 years, with peak onset between 10–14 years [7] while T2DM, on the other hand, is mostly associated with late onset, above 40 years.

These chronic complications lower the quality of life and cause premature death in patients with diabetes. Diabetic ketoacidosis was the most prevalent complication (23.1%) among early‐onset diabetes patients. A review of the literature identified diabetic ketoacidosis as the most prevalent complication (62.5%) among children with diabetes [10] which supports our results. It is estimated that more than 64% of children hospitalised for diabetic ketoacidosis may develop acute kidney injury. This highlights the prevalence and health impact of diabetic ketoacidosis in paediatric diabetic populations [23]. Diabetic ketoacidosis is a serious complication of diabetes; if not promptly and properly managed, it may result in death [11, 24]. It accounts for 50% of all deaths in the early‐onset diabetes population [11]. A mortality rate of 5.8% (3 out of 52 patients) was reported among our study participants; however, the exact cause of death was not known. Other complications of EOD include nephropathy, retinopathy, and neuropathy, which are associated with microvascular complications [25]. It is worth noting that clinical evidence for diabetes‐related microvascular complications is rare in early‐onset diabetes cases [25]. From our data, diabetic nephropathy prevalence was estimated at 3.8% and retinopathy at 7.7%. The low prevalence of nephropathy found in this study is in tandem with the 2.3% found among US paediatric diabetes cases [25]. However, other studies have reported higher prevalence of 13.4%–32.9% [10, 26]. While the prevalence of nephropathy found here may not be as high, it is a cause of concern. Nephropathy is a progressive condition that can lead to severe outcomes such as chronic kidney disease if left unmanaged, and this may negatively impact the quality of life of children. So, screening for microvascular complications is important to identify their occurrence at an early stage. Also, improving access to diabetic nephropathy screening using novel screening methods may help improve the detection and early treatment of diabetic nephropathy.

In our study, the type of diabetes was significantly associated with the development of complications (*p* < 0.05). This implies that the type of diabetes a patient has significantly influences the likelihood of developing complications. Secondary diabetes (53.8%) and T1DM (19.2%) were the major contributing factors to the development of complications. Several other studies have also reported a significant association between the type of diabetes and the development of complications [27, 28, 29]. These complications can significantly impact quality of life and increase mortality risk. There is a need for regular and comprehensive screening for complications among patients with EOD.

Literature on EOD from Ghana shows that most children living with T1DM face several challenges ranging from the lack of specialised diabetic clinics, a shortage of well‐trained health professionals, limited diagnostic devices to expensive treatment [30]. The healthcare system in Ghana is organised into three levels: tertiary (teaching hospitals), secondary (regional and sub‐regional hospitals); and primary (health centres and posts). Tertiary and secondary health facilities have more resources for clinically managing children with diabetes; however, they are not accessible to the majority of the population [31]. The primary health centres in Ghana, such as Community‐based Health Planning and Services (CHIPS) compounds and health centres, which are more accessible, mostly lack the equipment, trained staff, and other resources that are essential for managing these children. This leads to late diagnosis and a delay in diabetic management, which may result in diabetic complications [32]. Furthermore, since Ghana's National Health Insurance Scheme does not fully cover the management of diabetes, many families are unable to afford consistent management of the condition [32]. Children with diabetes and their families also suffer stigmatisation in society [33] and have mental health challenges. From Ghana, it was reported that psychosocial disorders, including stress, anxiety, and depression, are some common mental conditions that affect these families [30].

## 5 Clinical Management of Early Onset Diabetes in Ghana

Although the retrieved clinical data did not include specific details on how the cases were diagnosed, the Ghana National Guidelines for diabetes diagnosis recommend that T1DM and T2DM diabetes be diagnosed based on the following criteria: fasting plasma glucose ≥ 7.0 mmol/L, random plasma glucose ≥ 11.0 mmol/L, or HbA1c ≥ 6.5%, with or without the presence of ketonemia. The guidelines also recommend a glycemic target of HbA1c < 6.5%. It is worth noting that the healthcare facilities at the study site are not resourced to conduct genetic investigations and other expensive monogenic diabetes diagnoses; hence, the patients were not diagnosed with any form of monogenic diabetes. The 2023 Ghana National Guidelines for the Management of diabetes Mellitus outline both non‐pharmacological and pharmacological strategies for managing EOD mellitus and its complications, emphasising a multidisciplinary approach [34]. Non‐pharmacological management includes Medical Nutrition Therapy with personalised dietary plans for macronutrient balance and weight management, regular physical activity, age‐specific patient education, and self‐monitoring of blood glucose. T1DM management involves long‐acting basal insulin combined with rapid‐acting insulin at mealtimes. In T2DM, metformin is the first‐line oral glucose‐lowering agent, with insulin reserved for severe cases or acute illnesses. Acute complications such as hypoglycemia and diabetic ketoacidosis are managed through fluid resuscitation, electrolyte correction, insulin infusion, and prompt glucose or glucagon administration. Regular reviews every 3–6 months, along with statins and antihypertensives for co‐morbidities like dyslipidemia and hypertension, are also emphasised [34].

The guidelines from the Ministry of Health in Ghana also address rare and atypical forms of EOD, such as Monogenic Diabetes, including Maturity‐Onset Diabetes of the Young (MODY), Neonatal Diabetes, and Mitochondrial Diabetes. Early identification requires clinical suspicion based on family history, atypical presentation, and genetic testing [34]. Lifestyle modifications and sulfonylureas are the first‐line therapy for some MODY subtypes, while insulin therapy is needed in other forms. In mitochondrial diabetes, hearing aids or cochlear implants are recommended for managing associated hearing loss. Other rare genetic diabetes forms linked to insulin resistance, such as Rabson‐Mendenhall syndrome, and syndromic diabetes related to genetic disorders like Down syndrome require individualised, multidisciplinary care. The management goals include personalised treatment plans, genetic counselling, and team‐based care to improve clinical outcomes [34].

## 6 Limitations

Genetic testing for monogenic diabetes is not available at the clinics within the study site; therefore, patients included in this study were not screened for genetic causes of diabetes. Although clinicians may consider the possibility of familial diabetes during clinical evaluation, such investigations are not systematically recorded in the health system database. As a result, determining the underlying causes of secondary diabetes in these cases was challenging.

The Fisher Exact Test was employed due to the small sample size, which is reflective of the rarity of EOD compared to late‐onset diabetes. While we acknowledge the test's limited statistical power, it provides accurate, exact *p*‐values for small or unbalanced categorical datasets without relying on large‐sample assumptions. Additionally, although regression analysis could offer a more robust method to control for potential confounding variables, the dataset lacked the necessary variables to support such an analysis. These limitations are important and were considered in interpreting the study's findings.

## 7 Implications for Health Policy

To enhance the early detection and treatment of atypical diabetes in Ghana, health authorities should revise national guidelines to incorporate clinical signs of monogenic and secondary diabetes and create referral systems to specialised centres with genetic testing capabilities. Equipping healthcare providers with the skills to identify unusual diabetes presentations and consistently record relevant family history is crucial. Improving patient data management and collaborating with research institutions to offer affordable genetic testing will also strengthen diagnostic precision. Furthermore, establishing a national registry for atypical diabetes cases would aid in monitoring, research, and more effective healthcare planning.

## 8 Conclusion

In conclusion, addressing EOD in Ghana requires a multifaceted approach to overcome significant challenges such as limited diagnostic tools, financial constraints, and inadequate healthcare resources. Misdiagnosis and delayed treatment, coupled with a lack of awareness and social stigmatisation, exacerbate the burden on affected children and their families, leading to poor health outcomes and psychosocial stress. Strengthening healthcare systems, increasing awareness, and implementing the 2023 National Guidelines with an emphasis on multidisciplinary care and genetic testing are essential to improving the diagnosis, management, and long‐term outcomes for children with EOD in Ghana.

## Author Contributions

Conceptualisation, S.M.A.; design, S.B., C.K., J.A.M., G.K.H., J.A., and S.M.A.; data collection, S.B., C.K., G.K.H., and J.A.; data analysis, S.M.A. and S.B., original draft preparation, S.B., C.K., J.A.M., and S.M.A.; writing review and editing, S.B., C.K., J.A.M., G.K.H., J.A., and S.M.A.; student supervision, G.K.H., J.A., and S.M.A. All authors contributed important intellectual content presented in this manuscript. All authors have read and agreed to the final version of the manuscript.

## Ethics Statement

Ethical approval was obtained from the Navrongo Health Research Centre, Institutional Review Board (NHRCIRB) (ethics approval ID: NHRCIRB624).

## Conflicts of Interest

The authors declare no conflicts of interest.

## Data Availability

The authors have nothing to report.

## References

[1] AMA ADA , “American Diabetes Association Releases 2023 Standards of Care in Diabetes to Guide Prevention, Diagnosis, and Treatment for People Living With Diabetes,” https://diabetes.org/newsroom/american‐diabetes‐association‐2023‐standards‐care‐diabetes‐guide‐for‐prevention‐diagnosis‐treatment‐people‐living‐with‐diabetes.

[2] K. Ogurtsova , L. Guariguata , N. C. Barengo , et al., “IDF Diabetes Atlas: Global Estimates of Undiagnosed Diabetes in Adults for 2021,” Diabetes Research and Clinical Practice 183 (2022): 109118, 10.1016/j.diabres.2021.109118. 34883189

[3] A. P. Kengne and A. Ramachandran , “Feasibility of Prevention of Type 2 Diabetes in Low‐ and Middle‐Income Countries,” Diabetologia 67, no. 5 (2024): 763–772, 10.1007/s00125-023-06085-1. 38355989 PMC10954968

[4] E. Ekpor , S. Akyirem , and P. Adade Duodu , “Prevalence and Associated Factors of Overweight and Obesity Among Persons With Type 2 Diabetes in Africa: A Systematic Review and Meta‐Analysis,” Annals of Medicine 55, no. 1 (2023): 696–713. 36821504 10.1080/07853890.2023.2182909 PMC9970251

[5] F. Assah and J. C. Mbanya , “Diabetes in Sub‐Saharan Africa,” in Diabetes Mellitus in Developing Countries and Underserved Communities (Springer International Publishing, 2017), 33–48.

[6] CDC , “National Diabetes Statistics Report,” (2024), https://www.cdc.gov/diabetes/php/data‐research/index.html.

[7] Y. Reddy , Y. Ganie , and K. Pillay , “Characteristics of Children Presenting With Newly Diagnosed Type 1 Diabetes,” South African Journal of Child Health 7, no. 2 (2013): 46–48.

[8] G. A. Gregory , T. I. G. Robinson , S. E. Linklater , et al., “Global Incidence, Prevalence, and Mortality of Type 1 Diabetes in 2021 With Projection to 2040: A Modelling Study,” Lancet Diabetes and Endocrinology 10, no. 10 (2022): 741–760, 10.1016/S2213-8587(22)00218-2. 36113507

[9] A. A. Yameny , “Diabetes Mellitus Overview 2024,” Journal of Bioscience and Applied Research 10, no. 3 (2024): 641–645.

[10] O. Ugege , P. K. Ibitoye , and N. M. Jiya , “Childhood Diabetes Mellitus in Sokoto, North‐Western Nigeria: A Ten Year Review,” Sahel Medical Journal 16, no. 3 (2013): 97–101.

[11] W. Dong , S. Zhang , S. Yan , Z. Zhao , Z. Zhang , and W. Gu , “Clinical Characteristics of Patients With Early‐Onset Diabetes Mellitus: A Single‐Center Retrospective Study,” BMC Endocrine Disorders 23, no. 1 (2023): 216, 10.1186/s12902-023-01468-2. 37814295 PMC10563342

[12] F. Jiang , J. Yan , R. Zhang , et al., “Functional Characterization of a Novel Heterozygous Mutation in the Glucokinase Gene That Causes mody2 in Chinese Pedigrees,” Frontiers in Endocrinology 12 (2021): 803992. 34956103 10.3389/fendo.2021.803992 PMC8695754

[13] J. Støy , D. F. Steiner , S.‐Y. Park , H. Ye , L. H. Philipson , and G. I. Bell , “Clinical and Molecular Genetics of Neonatal Diabetes due to Mutations in the Insulin Gene,” Reviews in Endocrine and Metabolic Disorders 11 (2010): 205–215. 20938745 10.1007/s11154-010-9151-3 PMC2974937

[14] Y. D. Öngen , E. Eren , Ö. Demirbaş , et al., “Genotype and Phenotype Heterogeneity in Neonatal Diabetes: A Single Centre Experience in Turkey,” Journal of Clinical Research in Pediatric Endocrinology 13, no. 1 (2021): 80–87. 32820876 10.4274/jcrpe.galenos.2020.2020.0093 PMC7947723

[15] P. Theofilou and D. D. Vlastos , “The Psychological Burden of Families Having Children With Diabetes: A Literature Review Focusing on Quality of Life and Stress,” (2023). 10.3390/children10060937 PMC10296993 37371169

[16] J. A. Alhaddad , N. A. Alshakes , and M. N. Aljasim , “Quality of Life Among Children With Type 1 Diabetes Mellitus in Alahsa: A Cross‐Sectional Study,” Cureus 15, no. 6 (2023): e40766. 37485197 10.7759/cureus.40766 PMC10362093

[17] L. J. Kristensen , N. H. Birkebaek , A. H. Mose , L. Hohwu , and M. Thastum , “Symptoms of Emotional, Behavioral, and Social Difficulties in the Danish Population of Children and Adolescents With Type 1 Diabetes‐Results of a National Survey,” PLoS One 9, no. 5 (2014): e97543, 10.1371/journal.pone.0097543. 24842772 PMC4026318

[18] E. Ameyaw , S. B. Asafo‐Agyei , S. Thavapalan , A. C. Middlehurst , and G. D. Ogle , “Clinical Profile of Diabetes at Diagnosis Among Children and Adolescents at an Endocrine Clinic in Ghana,” World Journal of Diabetes 8, no. 9 (2017): 429–435. 28989569 10.4239/wjd.v8.i9.429 PMC5612833

[19] I. O. Oluwayemi , O. A. Oyedeji , E. O. Adeniji , et al., “A Ten‐Year Review of the Pattern and Outcome of Childhood Diabetes in Two State Teaching Hospitals in South‐West Nigeria,” Diabetes, Metabolic Syndrome and Obesity: Targets and Therapy 13 (2020): 4051–4057, 10.2147/DMSO.S275987. 33149644 PMC7604912

[20] S. Liu , M. Leone , J. F. Ludvigsson , et al., “Association and Familial Coaggregation of Childhood‐Onset Type 1 Diabetes With Depression, Anxiety, and Stress‐Related Disorders: A Population‐Based Cohort Study,” Diabetes Care 45, no. 9 (2022): 1987–1993, 10.2337/dc21-1347. 35913075 PMC9472496

[21] C. Kamrath , J. Rosenbauer , A. J. Eckert , et al., “Incidence of Type 1 Diabetes in Children and Adolescents During the COVID‐19 Pandemic in Germany: Results From the DPV Registry,” Diabetes Care 45, no. 8 (2022): 1762–1771, 10.2337/dc21-0969. 35043145

[22] S. A. de Vries , C. L. Verheugt , D. Mul , M. Nieuwdorp , and T. C. Sas , “Do Sex Differences in Paediatric Type 1 Diabetes Care Exist? A Systematic Review,” Diabetologia 66, no. 4 (2023): 618–630. 36700969 10.1007/s00125-022-05866-4 PMC9947056

[23] B. E. Hursh , R. Ronsley , N. Islam , C. Mammen , and C. Panagiotopoulos , “Acute Kidney Injury in Children With Type 1 Diabetes Hospitalized for Diabetic Ketoacidosis,” JAMA Pediatrics 171, no. 5 (2017): e170020, 10.1001/jamapediatrics.2017.0020. 28288246

[24] D. Msanga , K. Reis , N. Kayange , et al., “Diabetic Microvascular Complications Among Children and Adolescents in Northwestern Tanzania: A Cross‐Sectional Study,” Annals of Global Health 86, no. 1 (2020): 43. 32346524 10.5334/aogh.2669 PMC7181947

[25] L. Li , S. Jick , S. Breitenstein , and A. Michel , “Prevalence of Diabetes and Diabetic Nephropathy in a Large US Commercially Insured Pediatric Population, 2002–2013,” Diabetes Care 39, no. 2 (2016): 278–284. 26681728 10.2337/dc15-1710

[26] M. Karguppikar , C. Oza , N. Shah , V. Khadilkar , K. Gondhalekar , and A. Khadilkar , “Prevalence of Nephropathy in Indian Children and Youth With Type 1 Diabetes Mellitus,” Journal of Pediatric Endocrinology & Metabolism 35, no. 5 (2022): 585–592, 10.1515/jpem-2021-0644. 35304981

[27] A. Amutha , U. Ranjit , R. M. Anjana , et al., “Clinical Profile and Incidence of Microvascular Complications of Childhood and Adolescent Onset Type 1 and Type 2 Diabetes Seen at a Tertiary Diabetes Center in India,” Pediatric Diabetes 22, no. 1 (2021): 67–74. 32333449 10.1111/pedi.13033

[28] D. Dabelea , J. M. Stafford , E. J. Mayer‐Davis , et al., “Association of Type 1 Diabetes vs Type 2 Diabetes Diagnosed During Childhood and Adolescence With Complications During Teenage Years and Young Adulthood,” JAMA 317, no. 8 (2017): 825–835, 10.1001/jama.2017.0686. 28245334 PMC5483855

[29] J. M. Lawrence , J. Divers , S. Isom , et al., “Trends in Prevalence of Type 1 and Type 2 Diabetes in Children and Adolescents in the US, 2001‐2017,” Journal of the American Medical Association 326, no. 8 (2021): 717–727, 10.1001/jama.2021.11165. 34427600 PMC8385600

[30] B. A. Owusu , P. Ofori‐Boateng , A. Forbes , and D. T. Doku , “Knowledge of Young People Living With Type 1 Diabetes and Their Caregivers About Its Management,” Nursing Open 10, no. 4 (2023): 2426–2438. 36448367 10.1002/nop2.1498 PMC10006669

[31] V. A. Essuman , N. N. Tagoe , J. Akpalu , et al., “Morbidity and Complications of Diabetes Mellitus in Children and Adolescents in Ghana: Protocol for a Longitudinal Study,” JMIR Res Protoc 10, no. 1 (2021): e21440, 10.2196/21440. 33404517 PMC7817364

[32] J. Kratzer , “Structural Barriers to Coping With Type 1 Diabetes Mellitus in Ghana: Experiences of Diabetic Youth and Their Families,” Ghana Medical Journal 46, no. 2 (2012): 39–45. 23661816 PMC3645143

[33] H. E. Seo , M. Kim , E. Y. Doo , and J. Choi , “Process of Diabetes Management in Young Adults With Type 1 Diabetes,” Western Journal of Nursing Research 42, no. 4 (2020): 278–285, 10.1177/0193945919860865. 31347471

[34] Ministry Of Health Ghana , “Ghana National Guidelines for the Management of Diabetes Mellitus,” (2023).

